# Vitamin E Concentrations in Adults with HIV/AIDS on Highly Active Antiretroviral Therapy

**DOI:** 10.3390/nu6093641

**Published:** 2014-09-15

**Authors:** Daniella J. Itinoseki Kaio, Patricia Helen C. Rondó, Liania Alves Luzia, José Maria P. Souza, Aline Vale Firmino, Sigrid Sousa Santos

**Affiliations:** 1 Department of Nutrition, Public Health School, University of São Paulo, Av. Dr. Arnaldo, 715, CEP-01246-904 São Paulo, Brazil; E-Mails: daniellinhait@yahoo.com.br (D.J.I.K.); lianialuzia@usp.br (L.A.L.); alinefir@gmail.com (A.V.F.); 2 Department of Epidemiology, Public Health School, University of São Paulo, Av. Dr. Arnaldo, 715, CEP-01246-904 São Paulo, Brazil; E-Mail: jmpsouza@usp.br; 3 Department of Medicine, Center for Biological and Health Sciences, Federal University of São Carlos, Rodovia Washington Luís, km 235, CEP-13565-905 São Carlos, Brazil; E-Mail: sigridsantos@uol.com.br

**Keywords:** alpha-tocopherol, vitamin E, micronutrients, HIV, HAART

## Abstract

HIV/AIDS patients are probably more predisposed to vitamin E deficiency, considering that they are more exposed to oxidative stress. Additionally, there are an extensive number of drugs in the highly active antiretroviral therapy (HAART) regimens that may interfere with vitamin E concentrations. The objective of this study was to compare serum concentrations of alpha-tocopherol in 182 HIV/AIDS patients receiving different HAART regimens. The patients were divided into three groups according to regimen: nucleoside analog reverse-transcriptase inhibitors (NRTIs) + non-nucleoside analog reverse-transcriptase inhibitors (NNRTIs); NRTIs + protease inhibitors + ritonavir; NRTIs + other classes. Alpha-tocopherol was assessed by high-performance liquid chromatography. Multiple linear regression analysis was used to evaluate the effects of HAART regimen, time of use, and compliance with the regimen on alpha-tocopherol concentrations. Alpha-tocopherol concentrations were on average 4.12 μmol/L lower for the NRTIs + other classes regimen when compared to the NRTIs + NNRTIs regimen (*p* = 0.037). A positive association (*p* < 0.001) was observed between alpha-tocopherol and cholesterol concentrations, a finding due, in part, to the relationship between liposoluble vitamins and lipid profile. This study demonstrated differences in alpha-tocopherol concentrations between patients using different HAART regimens, especially regimens involving the use of new drugs. Long-term prospective cohort studies are needed to monitor vitamin E status in HIV/AIDS patients since the beginning of treatment.

## 1. Introduction

Acquired immunodeficiency syndrome (AIDS) is characterized by progressive depletion and dysfunction of CD4 T cells, associated with the development of opportunistic infections and neoplasms. Several mechanisms are involved in this process, including chronic immune activation that disrupts T cell homeostasis and induces oxidative stress, with depletion of plasma antioxidant concentrations [1,2]. Therefore, deficiency of micronutrients, especially antioxidants, is a common condition in HIV-infected patients, which can occur in all stages of the disease [3].

Antioxidants are known to play a vital role in the immune system, reducing oxidative stress induced by the excessive production of reactive oxygen species [4]. Apparently, oxidative stress plays a critical role in the stimulation of HIV replication and in the development of immunodeficiency [1].

The introduction of highly active antiretroviral therapy (HAART) has significantly increased the life expectancy of patients infected with HIV. In fact, the primary goals for initiating therapy are to reduce HIV-associated morbidity, to restore and preserve immunological function, to suppress plasma viral load, and to prevent HIV transmission [5]. Treatment is indicated for patients with HIV-associated clinical manifestations (irrespective of CD4 T lymphocyte count and plasma viral load) and for patients with CD4 T lymphocyte count ≤350 cells/mm^3^. Since 2013, treatment is recommended for all HIV-infected patients with CD4 T lymphocyte count ≤500 cells/mm^3^ [5].

With the emergence of HAART and all its benefits, researchers and clinicians believed that oxidative stress could be decreased in HIV-infected patients. However, the effect of HAART on oxidative stress is controversial. A previous study has shown that HAART permits better control of viral load, consequently increasing the concentrations of antioxidants to normal levels [4]. However, in an important review of the literature, Tang *et al.* [6] raised the hypothesis that HAART does not reduce oxidative stress to expected levels.

Among antioxidants, vitamin E has been extensively studied over the past decades, mainly because of its ability to serve as a chain-breaking antioxidant, to prevent the propagation of lipid peroxidation, and to reduce free radical damage [7]. Vitamin E deficiency is related to a reduction in T cells, natural killer cells and phagocytic response, compromising the cell-mediated response and humoral immunity [8]. Nutritional status and factors that can cause oxidative stress, such as HIV infection, have been suggested to predispose to vitamin E deficiency [9].

According to Fawzi [8] and Monteiro *et al.* [10], the rapid progression of HIV seems to be related, among other factors, to a deficiency in vitamin E considering the immunostimulatory and antioxidant properties of this vitamin. Although limited by small sample sizes and short follow-up, evidence suggests that vitamin E may provide some benefit to patients receiving HAART by increasing lymphocyte viability or decreasing viral load and oxidative stress [11]. Additionally, there are an extensive number of drugs in the HAART regimens [2] that may interfere with vitamin E concentrations in HIV/AIDS patients.

To our knowledge, no study has compared vitamin E status among patients on different antiretroviral therapies. Therefore, the objective of the present study was to compare serum vitamin E concentrations in patients with HIV/AIDS receiving different HAART regimens.

## 2. Experimental Section

The clinical protocol of this study (COEP No. 1915/09 and 0113/09) was in accordance with the ethical guidelines of the National Health Council. All subjects gave informed consent to participate in the study.

### 2.1. Study Design

A cross-sectional study was conducted on 182 HIV-infected men and women ranging in age from 20 to 59 years and with CD4 T lymphocyte counts ≥200 cells/mm^3^, who had received stable HAART for at least 6 months [3,12]. The patients were seen at an AIDS Treatment Referral Centre located in São Paulo city, Brazil. Exclusion criteria were pregnancy, use of vitamin and mineral supplements, cancer, recent surgery, acute infections, motor deficits impairing physical examination, concomitant participation in a nutritional intervention study, unavailable laboratory test data, and mental conditions that could interfere with the patient’s ability to be interviewed. The patients were selected consecutively between May and December 2009 according to the order of scheduled routine medical examinations.

### 2.2. General Data

A questionnaire was applied for collection of demographic, socioeconomic, life style, clinical, biochemical and immunological data, as well as data regarding HAART. The results of the most recent laboratory tests of the patients (up to 6 months prior to the interview) were considered for analysis.

### 2.3. Assessment of Nutritional Status

Body weight and height were measured in duplicate. The nutritional status of the patients was assessed based on body mass index (BMI) and was classified according to WHO criteria [13].

### 2.4. HAART Regimens

The different HAART regimens used by the patients were divided into three groups and included at least three drugs: 2 nucleoside analog reverse-transcriptase inhibitors (NRTIs) and 1 non-nucleoside analog reverse-transcriptase inhibitor (NNRTI); 2 NRTIs and 1 protease inhibitor (PI) plus ritonavir; 2 NRTIs and other classes including fusion inhibitors, integrase inhibitors, entry inhibitors, and PIs plus these drugs. The other classes of antiretroviral drugs specified above were grouped together because of the small number of patients using these recent drug classes.

Compliance with HAART was defined based on the criteria of Nemes *et al.* [14] as the ingestion of at least 95% of the prescribed drugs during the last 3 days prior to the interview.

### 2.5. Biochemical and Immunological Tests

Peripheral blood samples were collected after a 12-h fast in a dimly lit room to prevent degradation of vitamin E. The samples were centrifuged immediately at 3000 rpm for 15 min (Fanem^®^, Excelsa Baby I (Fanem, São Paulo, Brazil) and stored in properly identified amber Eppendorf^®^ tubes (Eppendorf, Hamburg, Germany) at −80 °C until the time of analysis.

Considering that alpha-tocopherol is the most biologically active form of vitamin E [6], alpha-tocopherol concentrations were measured in plasma by high-performance liquid chromatography (HPLC) as described by Arnaud *et al.* [15]. After thawing, 200 μL of the sample was added to 200 μL ethanol and the mixture was vortexed. Next, 5 mL hexane was added and the samples were mixed and centrifuged at 700× *g* for 5 min. A 250-μL aliquot of each supernatant was collected, dried under a nitrogen stream, and resuspended in 200 μL of the mobile phase (70% acetonitrile, 20% methanol, and 10% dichloromethane). Next, 50 μL of the mixture was injected into an HPLC system (Shimadzu SCL-10AVP System Controller, Kyoto, Japan) equipped with a Rheodyne manual sample injector (IDEX, Lake Forest, CA, USA). The chromatograms were integrated using the Shimadzu Class VP software (Shimadzu, Kyoto, Japan), and calibration curves were constructed for the calculation of alpha-tocopherol concentrations. Separation was performed on a 5-μm HyperClone ODS C18 analytical column (Phenomenex, Torrance, CA, USA). The cut-off points used for alpha-tocopherol were <11.6 μmol/L (deficient levels), 11.6 to 16.2 μmol/L (low levels), and >16.2 μmol/L (acceptable levels) [16].

Total cholesterol and fractions and triglycerides were analyzed by the cholesterol oxidase-phenol ampyrone (CHOD-PAP) and glycerol phosphate oxidase-phenol ampyrone (GPO-PAP) colorimetric enzymatic methods, respectively. For samples showing triglycerides <400 mg/dL (4.52 mmol/L), serum low-density lipoprotein (LDL)-cholesterol concentration was estimated using the formula of Friedewald [17].

Subpopulations of CD4 T lymphocytes were analyzed qualitatively by flow cytometry (FACSCalibur, BD Biosciences, San Jose, CA, USA). Viral load was evaluated quantitatively by the branched DNA 3.0 method (Versant, System 340 bDNA Analyzer, Bayer, Robinson Township, PA, USA).

### 2.6. Statistical Analysis

Data were double entered using the EpiData 3.1 program (EpiData Association, Odense, Denmark) and analyzed with the Stata 10 program (Stata Corporation, College Station, TX, USA). Absolute and relative frequencies and measures of central tendency (mean and median) and dispersion (range and standard deviation, SD) were used for description of the sample.

Multiple regression analysis was performed to determine the effect of the explanatory variables (duration of the last HAART regimen, type of the last HAART regimen, HAART compliance) on the response variable (alpha-tocopherol concentration). Gender, age, education, per capita income, smoking status, physical activity, BMI, clinical comorbidities, HIV infection (years), total cholesterol and fractions, triglycerides, and CD4 T lymphocyte count were used as control variables.

Vitamin E is a liposoluble compound, which depends on lipoproteins for its transport in human plasma. Therefore, among other variables, total cholesterol was included as a control variable in the multiple regression analysis. The explanatory variables were entered into the multiple regression models irrespective of their *p* value. A level of significance of 0.05 was adopted in the final model.

### 2.7. Ethical Approval

The present study was approved by the Ethics Committees of the Public Health School (COEP No. 079/09) and School of Medicine (CAPPesq No. 0113/09), University of São Paulo. All subjects gave informed consent to participate in the study.

## 3. Results

The baseline characteristics of the study population are shown in Table 1.

**Table 1:** Characteristics of the study population (*n* = 182).

Variables	*n* (%)	Mean (SD)
**Gender**		
Female	69 (37.9)	
Male	113 (62.1)	
**Age (years)**		43.8 (6.6)
**Education (years)**		10.9 (3.7)
Per capita income (R$/month) *		890.0 (991.1)
**Smoking status**		
No	75 (41.2)	
Yes	59 (32.4)	
Ex-smoker	48 (26.4)	
**Physical activity**		
No	122 (67.0)	
Yes	60 (33.0)	
**Body mass index (kg/m^2^)**		24.2 (3.8)
Underweight (<18.5)	8 (4.4)	
Eutrophic (18.5–24.9)	98 (53.8)	
Overweight (25.0–29.9)	66 (36.3)	
Obese (≥30.0)	10 (5.5)	
**Clinical comorbidity**		
No	82 (45.1)	
Yes	100 (54.9)	
**HIV transmission**		
Sexual	135 (74.2)	
Blood	17 (9.3)	
Unknown	30 (16.5)	
**HIV infection (years)**		11.5 (4.1)
**Use of antiretrovirals (years)**		10.1 (3.3)
**Duration of the last HAART regimen (years)**		4.0 (2.7)
**Last HAART regimen**		
NRTIs + NNRTIs	86 (47.3)	
NRTIs + PIs + ritonavir	84 (46.2)	
NRTIs + other classes	12 (6.6)	
**HAART compliance**		
No	33 (18.1)	
Yes	149 (81.9)	
**Alpha-tocopherol (μmol/L)**		21.9 (6.9)
<11.6	12 (6.6)	
>11.6–16.2	22 (12.1)	
>16.2	148 (81.3)	
** Total cholesterol (mmol/L)		5.0 (1.0)
<5.17	112 (61.9)	
5.17–6.18	44 (24.3)	
>6.18	25 (13.8)	
** HDL-cholesterol (mmol/L)		1.2 (0.4)
<1.03	30 (16.6)	
1.03–1.55	96 (53.0)	
>1.55	55 (30.4)	
** LDL-cholesterol (mmol/L)		2.9 (0.9)
<2.59	130 (71.8)	
2.59–4.11	35 (19.3)	
>4.11	16 (8.8)	
** Triglycerides (mmol/L)		2.0 (1.2)
<1.69	91 (50.3)	
1.69–2.26	36 (19.9)	
>2.26	54 (29.8)	
** Undetectable viral load (<50 copies/mm^3^)	154 (85.1)	
*** CD4^+^ T lymphocytes (cells/mm^3^)	649 (279.9) Median = 592 (200–1746)	

* 1 US$ = 1.70 Real (R$); SD, standard deviation; HIV, human immunodeficiency virus; HAART, highly active antiretroviral therapy; NRTIs, nucleoside analog reverse-transcriptase inhibitors; NNRTIs, non-nucleoside analog reverse-transcriptase inhibitors; PIs, protease inhibitors; HDL, high-density lipoprotein; LDL, low-density lipoprotein; ** *n* = 181; *** median (minimum and maximum).

Most of the patients were males (62.1%). The mean age was 43.8 (±6.6) years and the patients had 10.9 (±3.7) years of schooling and a per capita income of R$ 890.00 (991.1). One third of the patients were smokers and a similar proportion reported no physical activity. According to BMI, more than 50% of the subjects were eutrophic, 36% were overweight, 5.5% were obese, and approximately 4% were underweight. Clinical comorbidities associated with metabolic alterations were observed in 54.9% of the patients and mainly included dyslipidemia, hepatitis, lipodystrophy, arterial hypertension, diabetes mellitus, and insulin resistance (data not shown). Infection with HIV was due to unprotected sexual relations in 74.2% of the interviewed subjects and the mean duration of HIV infection was 11.5 (±4.1) years. The mean time of use of the last HAART regimen was 4.0 (±2.7) years. Most patients used the combination of NRTIs + NNRTIs (47.3%) and NRTIs + PIs plus ritonavir (46.2%). With respect to compliance with the HAART regimen, 81.9% of the subjects reported to have consumed the antiretroviral drugs during 3 days before the interview.

Mean alpha-tocopherol concentration was 21.9 (±6.9) μmol/L. Deficient, low and acceptable concentrations of this vitamin were observed in 6.6%, 12.1% and 81.3% of the patients, respectively.

Mean total cholesterol and high-density lipoprotein (HDL)-cholesterol levels were within the normal range recommended for adults (total cholesterol: <200 mg/dL (5.2 mmol/L) and HDL-cholesterol: ≥40 mg/dL (1.0 mmol/L)). Mean LDL-cholesterol and triglyceride concentrations were above the levels recommended by the US Department of Health and Human Services (LDL-cholesterol: <100 mg/dL (2.6 mmol/L) and triglycerides: <150 mg/dL (1.7 mmol/L)) [18]. Elevated concentrations of total cholesterol, LDL-cholesterol and triglycerides were observed in 13.8%, 8.8% and 29.8% of the patients, respectively. Low concentrations of HDL-cholesterol were observed in 16.6%.

Viral load was undetectable in 85.1% of the sample. Mean CD4 T lymphocyte count (cells/mm^3^) was 649 (±279.9) and the median value was 592 (range: 200–1746). The laboratory test results of one patient were excluded because no recent tests could be obtained.

In the final multiple linear regression model, serum alpha-tocopherol concentrations differed significantly between two of the HAART regimens used (*p* = 0.037) (Table 2). Alpha-tocopherol concentrations were on average 4.12 μmol/L lower for the combination of NRTIs + other classes (fusion inhibitors, integrase inhibitors, entry inhibitors, and PIs plus these drugs) when compared to the standard regimen (NRTIs + NNRTIs). Additionally, a positive association (*p* < 0.001) was observed between serum alpha-tocopherol concentrations and cholesterol, with an average increase in this vitamin of 0.082 μmol/L per 1 mg/dL (0.026 mmol/L) increase in cholesterol. The HAART regimen, duration of the last HAART regimen, HAART compliance and total cholesterol explained 19% of the variation in alpha-tocopherol concentrations.

**Table 2:** Multiple linear regression model, considering alpha-tocopherol as the outcome variable (*n* = 182).

Variables	Coefficient	Standard Error	CI (95%) *		*p* Value
**Alpha-Tocopherol**					
**Last HAART regimen**					
NRTIs + NNRTIs (reference)	0				
NRTIs + PIs + ritonavir	0.209	0.999	−1.764	2.184	0.834
NRTIs + other classes	−4.126	1.957	−7.990	−0.261	0.037
**Duration of the last HAART regimen** (years)	−0.229	0.182	−0.588	0.130	0.210
**HAART compliance**					
Yes (reference)	0				
No	1.146	1.192	−1.207	3.499	0.338
**Total cholesterol** (mg/dL)	0.082	0.012	0.058	0.106	<0.001
**Constant**	−3.214	5.185	−13.451	7.023	0.536

*R*^2^ = 0.21; adjusted *R*^2^ = 0.19. * CI, confidence interval; HAART, highly active antiretroviral therapy; NRTIs, nucleoside analog reverse-transcriptase inhibitors; NNRTIs, non-nucleoside analog reverse-transcriptase inhibitors; PIs, protease inhibitors. Other classes: fusion inhibitors, integrase inhibitors, entry inhibitors, and PIs plus these drugs.

## 4. Discussion

The HAART era has led to a decline in mortality and in comorbidities related to HIV infection. However, concern with the quality of life and survival of HIV-infected individuals continues in view of the frequent occurrence of metabolic disorders [19] that might be associated with anatomical changes and oxidative stress [6].

The results of this study showed a decrease of 4.12 μmol/L (*p* = 0.03) in mean alpha-tocopherol concentrations in patients receiving the combination of NRTIs + other classes when compared to those using NRTIs + NNRTIs. These lower concentrations of alpha-tocopherol in the group using NRTIs combined with the other classes of antiretroviral drugs (most of them new drugs) may be related to indications of salvage therapy, intolerance or poor treatment compliance, previous use of inadequate regimens, and primary resistance (subjects carrying a resistant virus) [20,21]. Regression analysis also showed a significant association between alpha-tocopherol concentrations and total cholesterol. In the population studied, alpha-tocopherol increased on average by 0.082 μmol/L per 1 mg/dL (0.026 mmol/L) increase in cholesterol. This finding might be due, in part, to the relationship between liposoluble micronutrients and lipid profile. Vitamin E is a liposoluble compound, which depends on lipoproteins for its transport in human plasma. Absolute concentrations of alpha-tocopherol increase with the degree of hyperlipidemia [22].

In the pre-HAART era, more than 60% of HIV-positive patients commonly had vitamin and/or mineral deficiency, a fact suggesting that the infection itself leads to nutritional deficiencies [23]. However, there are very few studies in the literature investigating the relationship between antiretrovirals and antioxidants and, to our knowledge, no study has compared vitamin E status in patients receiving different HAART regimens. In an *in vitro* study, Munteanu *et al.* [24] suggested that PIs combined with early alpha-tocopherol supplementation may decrease the drug-induced atherosclerotic risk. Scevola *et al.* [25] reported that the prolonged use of HAART, particularly PIs, is likely to improve micronutrient status. Tang *et al.* [4] found higher antioxidant levels in subjects using combination therapy with PIs and nucleoside analogs compared to those receiving nucleoside analog monotherapy.

Deficient (<11.6 μmol/L) and low (11.6 to 16.2 μmol/L) concentrations of alpha-tocopherol were observed in 18.7% of the population studied. Mean concentrations of alpha-tocopherol were 21.62 (±6.33), 22.60 (±6.92) and 19.11 (±10.21) μmol/L in the different intervention groups (NRTIs + NNRTIs, NRTIs +PI and NRTIs + others class, respectively). One of the difficulties in comparing the results of studies evaluating alpha-tocopherol concentrations in HIV-infected patients is the arbitrary use of different cut-off values. Pontes Monteiro *et al.* [26] observed low alpha-tocopherol concentrations (<18.0 μmol/L) in 41.3% of patients receiving only double therapy (zidovudine + didanosine). The use of a higher cut-off value and the inclusion of therapy with only two antiretroviral drugs may explain these results. Tang *et al.* [27] demonstrated low alpha-tocopherol concentrations (<11.6 μmol/L) in 22% of men using only NRTIs. Rousseau *et al.* [28] and Falcone [29] reported low alpha-tocopherol concentrations (<6 mg/L or <13.9 μmol/L and <5.0 mg/L or <11.6 μmol/L) for 30% and 6% of subjects receiving HAART, respectively. In addition to the use of different vitamin E cut-off values, the fact that older subjects with lower CD4 T lymphocyte counts were included in the study of Rousseau *et al.* [28] impairs comparison of the results.

An additional problem is that serum concentrations of micronutrients may not reflect the true nutritional status of subjects infected with HIV. According to Tang *et al.* [6], interpretation of low blood micronutrient concentrations is difficult since the metabolism of infected patients is altered during the acute and chronic phases of infection, with redistribution of circulating concentrations. Other relevant factors are the variation in recommended micronutrient intake and the low reproducibility of oxidative stress markers [30].

Dyslipidemia, a metabolic disorder commonly observed in patients with HIV/AIDS, is characterized by increases in total cholesterol, LDL-cholesterol and triglycerides, and a reduction in HDL-cholesterol. Hypertriglyceridemia, for example, is frequently observed in HIV-infected patients [31]. Risk concentrations of total cholesterol, LDL-cholesterol, HDL-cholesterol and triglycerides were observed in 13.8%, 8.8%, 16.6%, and 29.8% of the subjects studied, respectively. The changes in the lipid profile of patients with HIV/AIDS are probably the result of a combination of causes, including viral infection, effects of HAART and genetic factors [32,33]. In addition to the dyslipidemia observed in these patients, the use of HAART itself is also associated with lipodystrophy, fat redistribution characterized by peripheral fat wasting (face, arms, buttocks or legs) and central abdominal fat accumulation [33], which may also influence the concentrations of fat-soluble vitamin E.

Finally, this study has some limitations: (1) the cross-sectional design which does not permit to establish cause-effect relationships; and (2) the lack of measurement of acute phase proteins, although acute infection was an exclusion criteria for selection of the patients.

## 5. Conclusions

HIV/AIDS patients in Brazil will probably benefit from vitamin E supplementation, especially those receiving HAART regimens consisting of NRTIs and other classes (fusion inhibitors, integrase inhibitors, entry inhibitors, and PIs plus these drugs). Long-term prospective cohort studies are needed to monitor vitamin E status in HIV/AIDS patients since the beginning of treatment to confirm our results.
